# Fournier’s Gangrene Complicating Hematologic Malignancies: a Case Report and Review of Licterature

**DOI:** 10.4084/MJHID.2013.067

**Published:** 2013-11-01

**Authors:** Giovanni D’Arena, Giuseppe Pietrantuono, Emilio Buccino, Giancarlo Pacifico, Pellegrino Musto

**Affiliations:** 1 Onco-Hematology and Stem Cell Transplantation Unit, IRCCS “Centro di Riferimento Oncologico della Basilicata”, Rionero in Vulture (Pz), Italy; 2 Surgical Department, “S. Giovanni di Dio” Hospital, Melfi (Pz), Italy; 3 Scientific Direction, IRCCS “Centro di Riferimento Oncologico della Basilicata”, Rionero in Vulture (Pz), Italy

## Abstract

Fournier’s gangrene (FG) is a rare but severe necrotizing fasciitis of the external genitalia that may complicate the clinical course of hematologic malignancies and sometimes may be the first sign of the disease. The clinical course of FG is very aggressive and the mortality is still high despite the improvement in its management. Early recognition of FG and prompt appropriate treatment with surgical debridement and administration of antibiotics are the cornerstone of the management of this very severe disease. A review of the scientific literature focusing on the topic of FG complicating hematologic disorders is reported

## Introduction

Fournier’s gangrene (FG) is a particular type of necrotizing fasciitis (NF) involving the external genitalia and perianal or perineal regions with an acute rapidly progressive and potentially fatal course.^1^ Despite the first description of this disease was probably made by Ippocrates,^2^ this disorder is associated to the name of Alfred Fournier, a Parisian dermatologist and venereologist who firstly reported on 5 cases of otherwise healthy young men with a rapidly progressive gangrene of the penis and scrotum without apparent cause.^3^

The term necrotizing soft tissue infection has more recently been proposed because of including all forms of the disease.^4^ In fact, necrotizing infection may involve all type of soft tissue while diagnostic and therapeutic approaches are similar, regardless the anatomic or depth of infection.^5^

FG is quite rare while mortality, despite advances in management, is still very high, ranging from 20 to 30%, depending on the comorbidities and the early surgical debridement and use of antibiotics.^6^ Indeed, a study analyzing English literature collected 1,726 cases of FG diagnosed from 1950 and 1999 worldwide and found that mortality was 16%.^7^ Surprisingly, a higher mortality rate is reported in the last years. In fact, an unpublished study cited by Mallikarjuna et al, collecting 3,297 cases from 1950 to 2007, reported a mortality rate of 21.1%.^6^ Moreover, the mortality of FG seems to be higher in developed countries, such as America and Europe, than in underdeveloped countries.^7^ Taken together, this data may reflect the impact on the pathogenesis of FG of antibiotic resistance due to the use/abuse of antibiotics in the last years.

FG may affects subjects from infancy to old age, either men and women (though less frequently). In the large series of FG patients reported by Eke the male:female ratio was 10:1.^7^ This is probably due to the fact that in women perineal region may better drain through vaginal secretions.

In Table 1 are listed some of the most frequently conditions associated to FG. Diabetes has been reported with a prevalence ranging from 32 to 66% of patients with FG, while alcholism showed a prevalence ranging from 25 to 66%.^8^–^11^ Small vessel disease, defective phagocytosis, diabetic neuropathy, immunosuppression and poor hygiene have been considered to explain the relatively high incidence of FG in these patients.^1^

## Pathogenesis and Clinical Presentation

Less than one fourth of cases of FG is now considered idiopathic.^1^,^8^ Colorectal sources (30–50% of cases), urogenital sources (20–40% of cases), cutaneous infections and local trauma (20% of cases) are frequently identified as the cause of FG. The infectious process is due, in the majority of cases, to multiorganism infection (spreptococcall and staphilococcal species, enterobacteriaceae, anaerobic organism, and fungi). FG involves firstly superficial and deep fascial planes of genitalia; subsequently it spreads along the anatomical facial planes and to overlying skin, while muscles are often spared. However, the early event of FG is a localized infection near to the portal entry followed by an obliterative endoarteritis with cutaneous and subcutaneous vascular necrosis, leading to local ischemia and further bacterial proliferation. Via Buck and Dartos fascia, the infection of superficial perineal fascia may then spread to penis and scrotum, and, via Scarpa fascia, to the anterior abdominal wall. Not surprisingly, testicular involvement is very rare because of testicular arteries originates directly from the aorta, with a blood supply independent from the affected region.

The clinical presentation of FG is quite variable and a sudden or insidious onset may be both seen, the latter being more rare. Usually the infection starts as a cellulitis adjacent to the portal of entry, commonly in the perineum or perineal region. A strong fetid odour always occurs. Scrotal swelling, erythema, purulence or wound discharge, crepitation of inflammed tissue (due to the presence of gas forming organisms), fever, and pain are also frequently seen. The patients can rapidly progress to sepsis and multiorgan failure, the most common cause of death.

Laor et al proposed a scoring system, the so-called FG severity index (FGSI), to predict the prognosis.^12^Table 2 summarizes the clinical and laboratory parameters used. A score >9 is associated with 75% probability of death. On the contrary, a score <9 increases the probability of survival to 78% More recently, Yilmasilar et al updated the FGSI by adding

## Diagnosis and Management

The diagnosis of FG is usually made clinically. However, imaging may be particularly helpful to identify the rapidly progressing necrotizing process and the severity of the prognosis, that relies on the rapidity of diagnosis and the intense emergency management.

**Figure 1:**
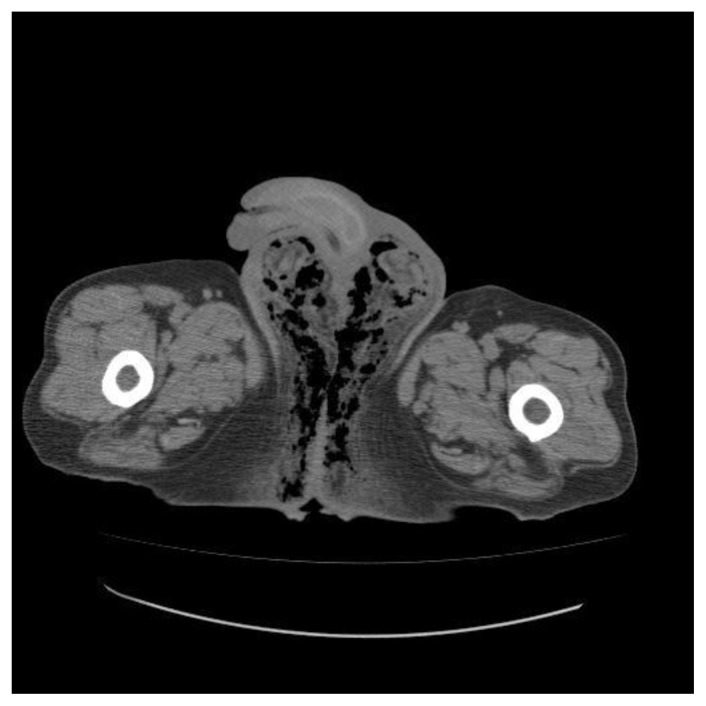
CT scan of a patient with FG showing emphysematous gangrene of perineum and scrotum (A). two additional parameters, such as age and extent of the disease.^13^

Computed tomography (CT) has a relevant importance in the diagnosis of FG because of its greater ability to identify and to evaluate the extent of the disease. CT scan can demonstrate asymmetric fascial thickening, coexisting fluid collection or abscess, fat stranding around the involved structures, and subcutaneous emphysema secondary to gas-forming bacteria (Figure 1).^14^

Plain radiography may shows air within the tissue, while ultrasonography is able to differentiate intrascrotal abnormalities, may shows thickhened and swolled scrotal wall, containing gas inside.^15^

Essentials of successfull management of patients with FG include early recognition of the disease, complete surgical debridement and early institution of appropriate broad-spectrum antibiotic therapy and supportive care for hemodynamic stabilization.

**Figure 2:**
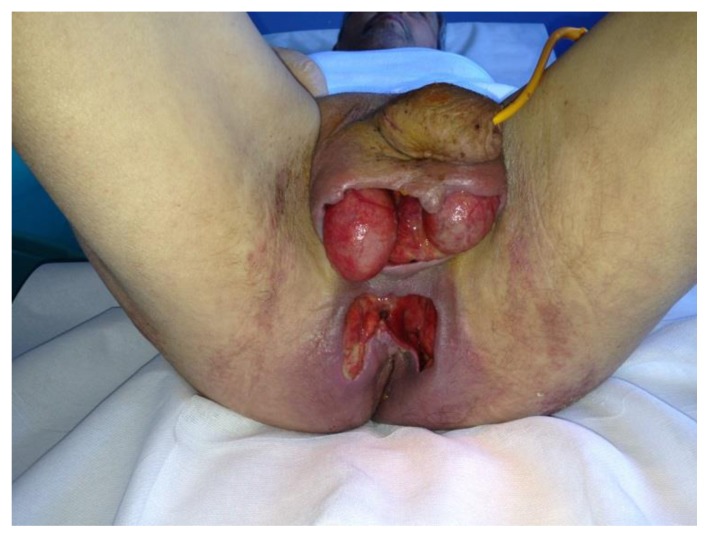
A case of FG after debridement with the complete excision of the necrotic tissue. The testicles are preserved.

Exploration and debridement must be undertaken as soon as possible.^16^,^17^ All necrotic tissue has to be removed (Figure 2). Because of testis are not involved, orchiectomy is performed rarely. Plastic reconstruction to provide skin cover may be useful to avoid infections and to accelerate the recovery.

Broad spectum antibiotic therapy, empiric initially and then adjusted when culture results are available, need to be instituted as soon as possibile. The combination of third generation cefalosporins or aminoglycosides, plus penicillin and metronidazole is the therapy of choice.^6^ The combinations of antibiotics should be effective against staphylococcal and streptococcal bacteria, gram-negative, coliforms, pseudomonas, bacteroides, and clostridium.^6^ Clindamycin, linezolide, daptomycin, tigecycline, and carbopenem may be also used.

Finally, hyperbaric oxygen therapy may also be used in some selected cases, despite this therapeutic approach is still matter of debate.^18^

FG complicating hematologic disorders

We performed a review of the scientific literature focusing on the topic of Fournier’s gangrene complicating hematologic malignancies. We searched the MEDLINE database using combinations of the following keywords: Fournier’s gangrene, hematologic malignancies, acute lymphoblastic leukemia, acute myeloide leukemia, chronic lymphocytic leukemia, chronic myeloid leukemia.

To the best of our knowledge, only 35 cases of FG associated to hematologic malignancies have been described to date (Table 3).^19^–^40^ The mean age was 35 years (range 6–83 years). Thirty (86%) patients were male, while only 3 patients (14%) were female. The large majority of them (88% of cases) were complications of previously diagnosed hematologic tumors, while only in 3 cases of acute myeloid leukemia (AML) and in 1 case of NHL FG was the first sign of the diseases.^24^,^29^,^35^ Twenty-two patients (63% of all cases) had AML; among these, 13 had acute promyelocytic leukemia (APL), a unique subtype of AML classified as AML-M3 in the French-American-Bristish classification system, that has distinctive morphological, biological and clinical features. In 12 of these cases FG was seen as a possible complication of all-trans retinoic acid (ATRA) administration, a treatment able to induce high rates of complete remission and cure when used alone or in combination with cytotoxic treatments in APL.^41^ ATRA induce terminal differentiation of abnormal promyelocytes by activation of RARα and by inducing degradation of promyelocytic leukemia (PML)/RARα.^42^ ATRA treatment is generally well tolerated. However, ATRA may cause leukocytosis and pulmonary complications, especially when used alone, with the possible development of the so-called “ATRA syndrome”.^43^,^44^ In addition, some other major side-effects can occur, such as dryness of skin, liver dysfunction, hyperlipidemia, bone pain, headache, fever, pseudotumor cerebri and Sweet’s syndrome. Scrotal ulcerations and necrotizing vasculitis may also be seen.^25^,^45^ It is reasonable to hypothesize that such lesions may progress to FG because of sovrainfections. Moreover, neutropenia may play a pathogenetic predisposing role. For those reasons, physicians must be aware that APL patients undergoing treatment with ATRA presenting with skin lesions may had necrotizing vasculitis needing carefully evaluation and prompt therapeutic approach.

Lymphoid malignancies, such as acute lymphoid leukemia (ALL), non-Hodgkin’s lymphoma (NHL) and Hodgkin’s lymphoma (HL), may be also complicated by FG that rarely, however, as reported above, may be the first sign of the disease.^35^

Moreover, FG has been observed in 2 patients with AML who underwent allogeneic bone marrow transplantation (allo-BMT), 2 patients with AML who underwent autologous (auto)-BMT, and one boy with refractory AML who underwent unrelated cord blood stem cell transplantation (CBT).^23^,^31^ All patients developed FG during the severe leukopenic phase of transplant (ranging from day +6 to day +25, this latter in unrelated CBT). Of interest, the two patients who underwent auto-BMT were young women aged 25 and 26-years old, respectively. Moreover, in all but one case *Pseudomonas aeruginosa* was isolated from blood and tissue cultures, despite FG is generally due to a mixed aerobic and anaerobic bacterial flora. It has been speculated that severe and prolonged granulocytopenia may contribute to the development of FG associated to *Pseudomonas aeruginosa* infection.^31^

Finally, only two cases of FG complicating a non malignant hematologic disorder, such as immune thrombocytopenia (IT), have been published so far. In the first case, a 68-year-old man with diabetes mellitus who was diagnosed with IT and treated with steroids and high-dose Ig without response. On day +36 he developed FG of perineal abscess.^46^*Klebsiella pneumonia* was cultured from the patient’s blood and necrotizing tissue. The second one was an otherwise healthy 66-year-old man, in which steroids and high-dose immunoglobulins were given with complete recovery of thrombocytopenia.^46^ However, on day +34, while on tapering steroids, FG developed as a consequence of perineal abscess. After debridement, antibiotics and reconstructive surgery FG completely resolved and pletelet raised to normal levels that still is maintained after 5 months of follow-up.

Overall, therapy for FG complicating hematologic disorders does not differ from that used in the case of FG associated to other diseases.

## Conclusions

FG is a rare necrotizing fasciitis of the perineum with a usually aggressive clinical course that may complicate hematologic malignancies and, sometimes, may presents as the first sign of the disease. Despite the progress in diagnosing and managing the disease, the mortality is still high, especially in hematologic patients with severe granulocytopenia. For that reason, early recognition of FG and prompt appropriate treatment have to be started as soon as possible. Finally, patients with APL receiving ATRA have to be monitored for signs and symptoms of FG because of the possibility of onset of onset of the appearance of genital ulcers and the onset of this severe complication.^48^

## Figures and Tables

**Table 1:** Most common comorbidities as risk factors for FG

✓ Diabetes ✓ Alchoholism ✓ Obesity ✓ Low socioeconomic status ✓ Acquired immune deficiecny syndrome (AIDA) ✓ Malignancies ✓ Malnutrition ✓ Prolonged hospitalization for debilitating illness ✓ Liver disease as cirrhosis ✓ Chemotherapy ✓ Intravenous drug use ✓ Iatrogenic immunosuppression (i.e., chronic corticosteroid use) ✓ Systemic lupus erythematosus ✓ Crohn disease

**Table 2:** Fournier’s Gangrene Severity Index (FGSI)

Variable/Score	4	3	2	1	0 (Normal values)
Temperature (°C)	>41	39–40.9	-	38.5–38.9	36–35.9
Heart rate (beats/min)	>180	140–179	110–139	-	70–109
Respiratory rate (breaths/min)	>50	35–49	-	25–34	12–24
Serum sodium (mmol/L)	>180	160–179	155–159	150–154	130–149
Serum potassium (mmol/L)	>7	6–6.9	-	5.5–5.9	3.5–5.4
Serum creatinine (mg/100 mL)	<3.5	2–3.4	1.5–1.9	-	0.6–1.4
(x 2 for acute renal failure)					
Hematocrit (%)	<60	-	50–59.9	46–49.9	30–45.9
White blood cell count (/mm^3^ × 1000)	>40	-	20–39.9	15–19.9	3–14.9
Serum bicarbonate (mmol/L)	>52	41–51.9	-	32–40.9	22–31.9

Extent of the disease (dissemination score): FG confined to the urogenital and/or anorectal region, add 1: FG confined to the pelvic region, add 2; FG extended beyond the pelvic region, add 6. Age score: age ≥60 years add 1, while age < 60 years, add 0. This score, proposed in 1995 by Laor et al [12], is based on deviation from reference ranges of the above parameters. Age and extent of disease have been added in 2010 by Yilmazlar et al [13] to improve the prognostic ability of FGSI. Each parameter is scored between 0 and 4, where the higher value indicates the greater deviation from normal. Score >9 correlates with increased mortality.

**Table 3:** Published cases of patients with hematologic malignancies and FG

Reference	Age	Sex	Disease	First sign/Complication	Note	Isolated bacteria	Specific therapy for FG	Prognosis of FG
Patrizi et al^19^	21	M	APL	Complication	Pre-ATRA era	P.aeruginosa	Surgery, topical anti septic	Good

Joo et al^20^	44	M	ALL	Complication		Not performed	Patient died within hours of hospital admission.	Died

Berg et al^21^	16	M	DLBCL	Complication		P.aeruginosa	Surgery, penicillin and clindamycin.	Died
	25	M	AML	Complication		P.aeruginosa	Gentamicin, carbenicillin, irradiation, surgery.	Died

Radaelli et al^22^	37	M	AML	Complication		P. rettgeri, P. aeruginosa.	Gentamicin, colistin, surgery.	Good
	14	M	ALL	Complication		Negative tests	Colistin, carbenicillin, chloramphenicol, lincomycin, surgery.	Died
	19	M	NHL	Complication		P.aeruginosa	Colistin, amikacin, surgery.	Good
	20	M	ALL	Complication		P.aeruginosa	Ceftazidime, amikacin, surgery, hyperbaric oxygen therapy.	Good

Martinelli et al^23^	41	M	AML		Allogeneic BMT	P.aeruginosa	Imipenem, surgery.	Good
	26	F	AML		Allogeneic BMT	P.aeruginosa	Amikacin, surgery	Good

Faber et al^24^	50	M	APL	Firs sign		E.coli	Clindamycin, penicillin G, ciproxin, surgery.	Died

Paydas et al^25^	26	M	APL	Complication	ATRA therapy ATRA therapy	Not found	Ceftriaxone, teicoplanin, amphotericin B (to treat unknown origin fever).	Good
	26	F	APL	Complication		Not reported	Ceftazidime, amikacin, clindamycin.	

Levy et al^26^	44	M	APL	Complication		S. faecalis, S. coagulase negative	Piperacillin/tazobactam, netilmicin, vancomycin, amphotericin B, metronidazol, surgery.	Died

Goto et al^27^	43	M	APL	Complication	ATRA therapy	Not reported	Surgery, antibiotics therapy (not otherwise specified)	Good

Yumura et al^28^	83	F	DLBCL	Complication		Not reported	Surgery	Good

Islamoglu et al^29^	33	M	AML	First sign		Bacteroides fragilis	Surgery; antibiotics (not otherwise specified)	Died

Castellini et al^30^	54	M	HL	Complication		Not reported	Surgery, metronidazole, gentamycin, hyperbaric oxygen therapy	Good

Yoshida et al^31^	16	M	AML	Complication	CB transplantation	P.aeruginosa	Broad spectrum antibiotics (not otherwise specified)	Died

Fukuno et al^32^	43	M	APL	Complication	ATRA therapy ATRA therapy ATRA therapy ATRA therapy	Not Reported	‘Antibiotic ointment’ (not otherwise specified) in the first two cases and steroid in the last two patients.	Good
				
	52	M	APL	Complication				Good
	53	F	APL	Complication				Good
	41	F	APL	Complication				Good

Bakshi et al^33^	10	M	T-ALL	Complication		P.aeruginosa	Ceftazidime, imipenem/cilastatin, surgery	Good
	6	M	AML	Complication		P.aeruginosa	Imipenem/cilastatin	Good
	9	M	NHL	Complication		Not reported	Imipenem, surgery	Good

Mantadakis et al^34^	21	M	ALL	Complication		P.aeruginosa	Meropenem, piperacillin/tazobactam, metronidazole, linezolid, voriconazole, colistin, surgery	Died

Lohana et al^35^	70	M	Mycosis fungoides	First sign		Staphylococcus aureus, E. coli, Group B streptococci, mixed anaerobes	Vancomycin, meropenem, surgery	Good

Zhou et al^36^	49	M	T-NHL	Complication	Cutaneous (penis) T- NHL	Not reported	Interferon and ultraviolet B, surgery	Good

Naithani et al^37^	23	M	APL	Complication	ATRA therapy ATRA therapy ATRA therapy	Not reported	Local clotrimazole powdern (in all cases). The second and third cases received ‘appropriate antibiotics’ (not otherwise specified)	Good
	17	M	APL	Complication		Not reported		Good
	17	M	APL	Complication		S.aureus, E.coli		Good

Oiso et al^38^	51	M	AML	First sign		Corynebacterium spp.	Cefpirome, clindamycin, cilastatin	Good

Kaya et al^39^	71	M	NHL	Complication	Intravascular NHL	P.aeruginosa	Surgery; antibiotics (not otherwise speified)	Good

Durand et al^40^	53	M	AML	Complication		Rhizopus microsporus	Piperacillin-tazobactam, meropenem, amphotericin B, micafungin, surgery	Died

Legend: M: male; F: female; AML: acute myeloid leukemia; APL: acute promyelocytic leukemia; NHL: non-Hodgkin’s lymphoma; DLBCL; diffuse large B-cell lymphoma; HL: Hodgkin’s lymphoma; ALL: acute lymphoblastic leukemia; ATRA;: all trans retinoic-acid; CB: cord blood; BMT: bone marrow transplantation
